# Annual flower strips support pollinators and potentially enhance red clover seed yield

**DOI:** 10.1002/ece3.4330

**Published:** 2018-07-16

**Authors:** Maj Rundlöf, Ola Lundin, Riccardo Bommarco

**Affiliations:** ^1^ Department of Biology Lund University Lund Sweden; ^2^ Department of Entomology and Nematology University of California Davis California; ^3^ Department of Ecology Swedish University of Agricultural Sciences Uppsala Sweden

**Keywords:** biological control, *Bombus*, ecological intensification, ecosystem services, floral resources, habitat enhancement, pollination, *Protapion*, red clover, *Trifolium pratense*

## Abstract

Ecological intensification provides opportunity to increase agricultural productivity while minimizing negative environmental impacts, by supporting ecosystem services such as crop pollination and biological pest control. For this we need to develop targeted management solutions that provide critical resources to service‐providing organisms at the right time and place. We tested whether annual strips of early flowering phacelia *Phacelia tanacetifolia* support pollinators and natural enemies of seed weevils *Protapion* spp., by attracting and offering nectar and pollen before the crop flowers. This was expected to increase yield of red clover *Trifolium pratense* seed. We monitored insect pollinators, pests, natural enemies and seed yields in a total of 50 clover fields along a landscape heterogeneity gradient, over 2 years and across two regions in southern Sweden. About half of the fields were sown with flower strips of 125–2,000 m^2^. The clover fields were pollinated by 60% bumble bees *Bombus* spp. and 40% honey bees *Apis mellifera*. The clover seed yield was negatively associated with weevil density, but was unrelated to bee species richness and density. Flower strips enhanced bumble bees species richness in the clover fields, with the strongest influence in heterogeneous landscapes. There were few detectable differences between crop fields with and without flower strips. However, long‐tongued bumble bees were redistributed toward field interiors and during phacelia bloom honey bees toward field edges. Clover seed yield also increased with increasing size of the flower strip. We conclude that annual flower strips of early flower resources can support bumble bee species richness and, if sufficiently large, possibly also increase crop yields. However, clover seed yield was mainly limited by weevil infestation, which was not influenced by the annual flower strips. A future goal should be to design targeted measures for pest control.

## 1 INTRODUCTION

The demand for agricultural products is estimated to increase dramatically, and there is a need to develop agricultural management practices that secure production while minimizing negative environmental impacts (Tilman, Balzer, Hill, & Befort, 2011). Ecological intensification provides options to replace external inputs with ecosystem services generated within the agroecosystems (Bommarco, Kleijn, & Potts, 2013). Crop pollination (Klein et al., 2007) and biological pest control that limit crop losses (Losey & Vaughan, 2006), provided by a great number of beneficial pollinating and predatory insects, are two key ecosystem services in this context.

A challenge is that land use conversion into intensive agriculture has led to losses of habitat and resources for beneficial organisms, with negative consequences for ecosystem services (Kennedy et al., 2013; Rusch et al., 2016). Lack of arable land globally denies the option to convert large areas of cropped land into perennial habitat, such as permanent grasslands, generally suitable for predators and pollinators (e.g., Garibaldi et al., 2011). More targeted and economic ways to increase the number of beneficial organisms and enhance the protection and pollination of crops are needed (Bianchi, Ives, & Schellhorn, 2013; Garibaldi et al., 2014). In particular, at critical times in the season, the right resources need to be added (e.g., van Rijn & Wäckers, 2016) that currently form bottle‐necks for population growth of these organisms in intensively cropped landscapes (Rundlöf, Persson, Smith, & Bommarco, 2014; Schellhorn, Parry, Macfadyen, Wang, & Zalucki, 2015). Importantly, the supporting measure needs to be evaluated with consideration of the landscape context (Carvell et al., 2011; Kleijn, Rundlöf, Scheper, Smith, & Tscharntke, 2011), and it should also be aligned with mainstream crop production systems, practicable for the farmer to implement, not enhance pest pressure and provide enough yield gain to compensate for area lost from production.

An often suggested, but poorly tested, option is to add early season food resources by sowing strips with early blooming flowers that provide pollen and nectar in the landscape (Westphal, Steffan‐Dewenter, & Tscharntke, 2003; Williams, Regetz, & Kremen, 2012). An indication that adding early season resources can be an effective measure is increases in population growth and size of bumble bee colonies with the presence of early flowering oilseed rape in the landscape (Persson & Smith, 2013; Westphal, Steffan‐Dewenter, & Tscharntke, 2009). Sowing strips with wild flowers is an increasingly tested measure to support beneficial insects. Typically the strip is sown with multiple species designed to provide resources and shelter across the season. Several recent examples show benefits of this intervention for pollinating insects and crop pollination (Blaauw & Isaacs, 2014; Carvalheiro, Seymour, Nicolson, & Veldtman, 2012), and for natural enemies to pests and crop protection (Jonsson, Wratten, Landis, Tompkins, & Cullen, 2010; Tschumi, Albrecht, Entling, & Jacot, 2015). Rarely have, however, the effects of introducing a seasonally timed monoculture flower resource on pollinators, pests, natural enemies and crop yield been considered simultaneously.

A potential problem is that the added habitat competes with the crop for the ecosystem service providers. Although normally much smaller in size compared with the crop, the added resource might be attractive to, for instance, pollinating insects that are drawn away from the crop. Cross‐habitat movement is known to impact resource flows and population dynamics of interacting organisms (Polis, Anderson, & Holt, 1997; Smith et al., 2014). For agricultural landscapes there has been a focus on the distribution and interactions of organisms between crop and natural habitats (Rand, Tylianakis, & Tscharntke, 2006). For instance, flower visiting insects can be pulled away from a natural habitat by adjacent mass‐flowering crops, negatively affecting wild plant pollination (Holzschuh, Dormann, Tscharntke, & Steffan‐Dewenter, 2011). Less is understood on how organisms flow between crops (but see Bommarco & Fagan, 2002; Riedinger, Renner, Rundlöf, Steffan‐Dewenter, & Holzschuh, 2014) and other managed habitats such as flower strips, and whether adding a highly attractive resource adjacent to the crop risks pulling away service‐providing species from the target crop (but see Lundin et al., 2017).

Here we explored whether adding a flower resource, which starts to flower before and then co‐flowers with the target crop, affects species richness and abundance of pollinating insects, pest abundance and biological control, and yield. Annually sown strips of early blooming phacelia *Phacelia tanacetifolia* were established on the edge of crop fields sown with later blooming red clover *Trifolium pratense* for seed production. Insect pollinators, pests, natural enemies and seed yields were monitored in 50 clover fields with or without strips situated in simple intensively cropped to heterogeneous landscapes. We tested the hypotheses that (a) seed yield is positively related to pollinator abundance and negatively to pest abundance; (b) early blooming flower strips can enhance yield by supporting pollination and biological control of pests (measured as parasitism rate) in a later blooming crop; (c) the addition of a flower strip has a larger positive influence on pollination and pest control in simple landscape because service providers lack other alternative resources; and (d) larger flower strips have a greater positive influence. To test if the flower strip distracted pollinators from the crop, we explored how pollinators distributed over the two habitats, flower strip and clover field, when both phacelia and clover flowered and we compared densities in fields with and without strips. We also tested if the pollinator communities shared similar distributions of tongue length, which is an important pollination trait (Garibaldi et al., 2015), and if the flower strip influenced short‐ and long‐tongued bumble bee differently.

## 2 MATERIALS AND METHODS

### 2.1 Experimental design

Production of red clover seeds depends on insect pollination for seed set, which is mainly performed by bumble bees (Free, 1993). Seed weevils are the most common pests and can cause great yield losses, but parasitic wasps attack them and can provide biological pest control (Lundin, Rundlöf, Smith, & Bommarco, 2012). Densities of pollinators, weevils and their parasitoids, and crop yield were estimated in 50 red clover seed fields in 2009 (24 fields) and 2010 (26 fields). Fields were located in the two regions of Skåne (28 fields) and Östergötland (22 fields) (Supporting Information Figure S1, Supporting Information Table S1). They were sown with several red clover cultivars differing in ploidy (diploid: 25 fields, or tetraploid: 25 fields), which has been shown to influence inflorescence number, seed set and yield (Vleugels, Roldan‐Ruiz, & Cnops, 2015). Fields were separated by at least 2.5 km within a year and had an average size of 9.1 ha (range 4–24 ha) (Supporting Information Table S1).

The landscapes surrounding the clover seed fields were characterized within a 1‐km radius using digital land use data from the Integrated Administration and Control System (IACS). Proportion of land covered by annual crops (51%, 14%–76% (mean, min–max)) and seminatural habitats (5.4%, 0.99%–18%) varied across landscapes, but the proportions of these habitat types did not differ between fields with a flower strip and control fields (Supporting Information Table S1).

Strips of phacelia ranging in size from 125 to 2,000 m^2^ were sown at the edge of 22 fields. Details on flower strip establishment are provided in the Supplementary Information. Flower strips were cut when the red clover had started flowering, but after we had completed at least one survey with both habitats flowering at the same time (see below).

### 2.2 Pollinator surveys

To determine how pollinators responded to the flower strips, bumble bee *Bombus* spp. workers and males were collected and bumble bee queens, honey bees *Apis mellifera*, solitary bees, butterflies and moths were registered along two 1 m wide and 50 m long transects in the clover fields. The two transects were located 8 and 100 m from the field edge, along the phacelia planting at fields with flower strips. For smaller fields, the interior transect was placed in the field center. Each clover field was visited three to five times (average 3.7 visits per field in 2009 and 4.0 in 2010) from late June to mid‐August, to cover the main flowering period of the red clover. To compare the pollinators between flower strips and clover fields, pollinator density was also surveyed in the strips along one 1 m wide and 50 m long transect. Each strip was surveyed 1–2 times on the same day as the clover field was surveyed, between late June and mid‐July. Pollinator surveys were conducted on days with warm (minimum 17°C), sunny and calm (5 or less on the Beaufort wind scale) weather. The collected bumble bees were determined to species in the laboratory following Løken (1973), Prys‐Jones and Corbet (1986), and Edwards and Jenner (2005) and using the reference collection at the Biological Museum, Lund University.

Flower abundance, in both the clover fields and flower strips, was registered at each pollinator survey, within the same transects. Four 0.5 × 0.5 m squares were placed with equal spacing along the 50 m transect and the number of flowering red clover inflorescences (>5 open flowers) and phacelia flowering plants (≥1 open flower) were counted within the squares.

### 2.3 Pests and natural enemies

To determine if the flower strips influenced the main crop pests and their parasitoid natural enemies, we measured the abundance of *Protapion* spp. weevils and parasitism rates provided by their natural enemy wasps in each field. Red clover inflorescences, at the morphological stage where all individual flowers had recently withered, were picked between late July and mid‐August for rearing of the weevils and their parasitoids. In 2009 60 inflorescences and in 2010 120–240 inflorescences (the higher number from fields belonging to farmers with low weevil densities in 2009) were picked from each of the two pollinator transects. The collected inflorescences were divided into batches of 30 inflorescences and put in cardboard boxes (20 × 7 × 7 cm). Insects were reared in the boxes and a connected transparent plastic tube was used to collect emerged weevils and wasps. We identified and counted the number of emerging *Protapion* spp. weevils per sample. Because newly hatched weevils lack some key morphological characters used for species identification, we were not able to determine them to species level. Based on pan trapping of the adults, we know, however, that the most common species which infest red clover inflorescences are *Protapion trifolii* and *Protapion apricans* (Lundin et al., 2012). Parasitic wasps were determined to two morphospecies, *Pteromalidae* spp. and *Triaspis* spp. which dominated the samples. Both of these contain several true species which parasitize clover seed weevils (Kruess & Tscharntke, 1994; Lundin et al., 2012). Other parasitic wasps were occasionally found in the samples and these were excluded because the host was unknown.

### 2.4 Seed yield

To determine if the flower strips influenced the clover seed yield (kg/ha), clover inflorescences on plants rooted within 1 m^2^ plots were manually harvested in eight plots per field, with four plots evenly spaced alongside each of the two pollinator transects. Harvesting was done just before the farmer harvested the field, between mid‐August and late September. Inflorescences were dried and threshed, and the seeds were rinsed and weighed at the Rural Economy and Agricultural Societies’ experimental farm at Sandby, Skåne.

### 2.5 Statistical analyses

We used general (PROC MIXED) and generalized (PROC GLIMMIX) linear mixed models in SAS 9.4 for Windows (SAS Institute Inc., Cary, NC) to analyze data, with values averaged (when assuming normal error distribution) or summed (when assuming Poisson or binomial error distribution) per transect prior to analyses. Field identity was included as a random factor, to accounting for transects located at the same site.

To determine if pollinators, pests and yields differed between transects within fields with flower strips and control fields without flower strips, we specified a model with presence of flower strip, year, region, transect, ploidy, field size, proportion of arable land in the 1 km circular landscape surrounding the clover field, and flower density as explanatory variables. Because inflorescence number covary with ploidy (Vleugels et al., 2015), we standardized flower density within ploidy level by subtracting the group mean. The model further included two‐way interactions between flower strip presence and year, region, transect, and proportion of arable land. Number of bumble bee species, community weighted mean (CWM) of bee tongue length, and yield of clover seed (kg/ha) were dependent variables modeled with normal error distribution. CWM of tongue length was calculated based on the abundance of bees identified to species or species group weighted by species‐specific tongue lengths (Supporting Information Table S2) (Díaz et al., 2007). Total number of bumble bees (bees per transect), number of short‐tongued (<7 mm) and long‐tongued (>7 mm) bumble bees (see Supporting Information Table S2) and honey bees were analyzed using generalized models with Poisson error distribution and log link. The ln‐transformed number of survey rounds was included as offset to account for the variation in survey intensity between fields. Number of weevils (including the parasitized) was analyzed using a generalized model with Poisson error distribution and log link with ln‐transformed number of collected clover inflorescences as offset. Number of parasitoids divided by the sum of weevils and parasitoids was analyzed using a generalized model with binomial error distribution and logit link. To determine if the flowering phacelia attracted bees away from the clover, we repeated the analyses comparing bee densities between fields with and without a flower strip, only including data from the time period when both the phacelia and clover flowered at sites with flower strips (see methods in Supporting Information).

For fields with flower strip, we analyzed if the size of the flower strip was related to pollinators, pests, and yields, using a model with year, region, transect, ploidy, and flower strip area standardized within ploidy as explanatory variables and the interaction between transect location and flower strip area. Even though the two levels of ploidy of the clover cultivar were similarly distributed over years and treatments (Supporting Information Table S1), the size of the flower strip differed between ploidy levels (*F*_1,20_ = 11.92, *p* = 0.0025); strips at fields with diploid clover were larger (1,431 m^2^, 1,154–1,708 [mean, 95% confidence limits]) than at fields with tetraploid clover (835 m^2^, 605–1,066). Since the two variables partly explained the same variation in the dependent variables, we standardized the flower strip area within ploidy by subtracting the group mean. The same dependent variables as before were used in this model: bumble bee species richness, and number of bumble bees, short‐ and long‐tongued bumble bees, honey bees and weevils, CWM tongue length, parasitism rate, and seed yield.

To determine the pollinator attractiveness of the phacelia in relation to the clover, bumble bee, and honey bee densities from surveys when both the two habitats flowered were related to transect: flower strip or edge or interior transect in the clover field. The model also included year, region, ploidy, field size, proportion arable land, and flower density as explanatory variables.

Finally, to test if pollinator and pest abundances explained clover seed yield, we specified a model with the average yield per transect as dependent variable, year, region, transect, ploidy, proportion arable land, field size, flower density, bumble bee species richness, short‐ and long‐tongued bumble bee densities, honey bee density (bees per transect) and weevil density (weevils per inflorescence) as explanatory variables.

Interactions between factors were examined using the SLICE option in the LSMEANS statement. Post hoc tests for where species richness differed between fields with and without flower strips along the arable land gradient and bee densities differed between transects were performed using the ESTIMATE option. Correlations among explanatory continuous variables were explored using PROC CORR (Pearson *r* < 0.42 in all cases) and collinearity by the variance inflation factor (VIF) in PROC REG (VIF <1.8 in all cases, indicating no collinearity; Zuur, Ieno, & Elphick, 2010). Distribution of residuals was assessed visually on plots and by Shapiro–Wilks tests, and homogeneity of variance between groups was assessed using Levene's test. When heterogeneous variances were detected, variances were modeled separately for the groups using a repeated (or random for PROC GLIMMIX) statement with the factor as the group factor. The estimation method was restricted maximum likelihood (REML) and the denominator degrees of freedom were estimated with the Kenward–Roger method or the containment method (Littell, Milliken, Stroup, Wolfinger, & Schabenberger, 2006).

## 3 RESULTS

### 3.1 Pollinators

**Figure 1:**
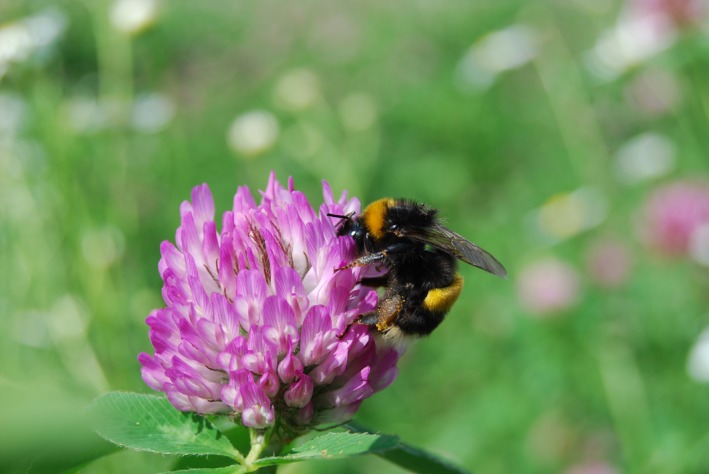
*Bombus terrestris* aggr. worker, the most common pollinator in red clover seed fields, visiting a red clover *Trifolium pratense* flower

During the 2 years of study, 6,786 pollinators were recorded visiting the red clover in the fields, 4,053 in 2009 (178 transects) and 2,733 in 2010 (210 transects). Sixty percent were bumble bees (Figure 1), 40% were honeybees and 24 individuals were solitary bees (<1%) (Supporting Information Table S2). The clover was also visited by 184 butterflies and 34 silver Y moths *Autographa gamma*, but these were not considered further.

**Figure 2:**
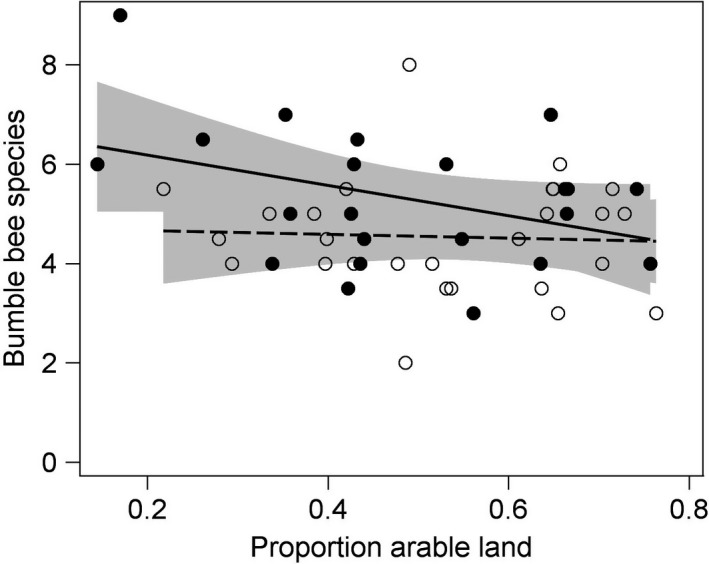
Bumble bee species richness per transect was higher in red clover seed fields with a phacelia flower strip (filled circles, solid line, *n* = 22 fields) compared with control fields (open circles, dashed line, *n* = 28 fields), but the difference was only evident at low proportions of arable land in the 1 km landscape surrounding fields. The shaded areas indicate 95% confidence limits for the linear relationships

**Figure 3:**
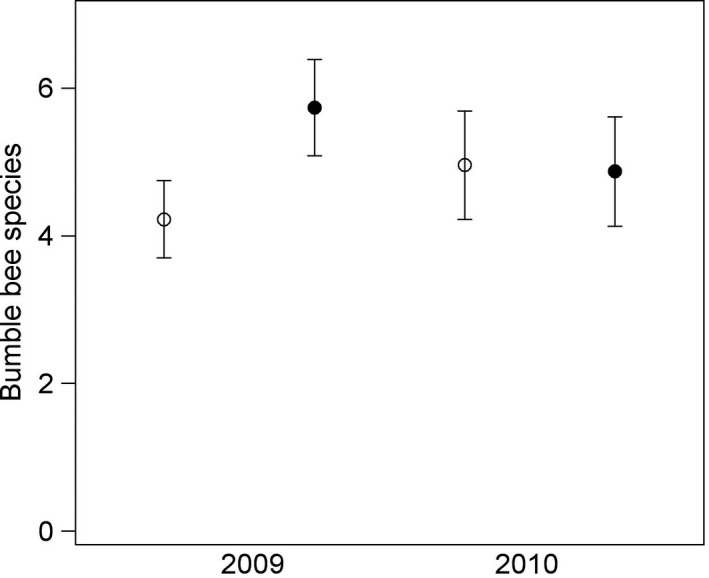
Bumble bee species richness per transect was higher in red clover seed fields with a phacelia flower strip (filled circles, *n* = 22 fields) compared with control fields (open circles, *n* = 28 fields) in 2009, but not 2010. Means (circles) and 95% confidence limits (error bars) are based on model‐estimated least square means

Overall, bumble bee species richness was 16% higher in clover fields with a flower strip, but there was an interaction between presence of flower strip and proportion arable land (Table 1, Figure 2). Post hoc tests gave that species richness was higher in fields with strips compared with control fields at 30% (*t*_33_ = 3.00, *p* = 0.0051), 40% (*t*_37_ = 3.01, *p* = 0.0047) and 50% (*t*_42_ = 2.31, *p* = 0.026) arable land in the surrounding landscape, but not at 60% (*t*_39_ = 0.78, *p* = 0.44) or 70% (*t*_34_ = −0.33, *p* = 0.75) arable land (Figure 2). There was also an interaction between presence of flower strip and year (Table 1), where flower strips enhanced species richness only in 2009 (*F*_1,14_ = 15.21, *p* = 0.0016) but not in 2010 (*F*_1,48_ = 0.03, *p* = 0.87) (Figure 3). Bumble bee species richness was not related to size of the flower strip, region, transect, ploidy, field size or flower density (Table 1).

**Table 1:** Bumble bee species richness, numbers (bees per transect) of all bumble bees, short‐tongued (<7 mm) and long‐tongued (>7 mm) bumble bees and honey bees and community weighted mean (CWM) bee tongue length in relation to year (2009, 2010), region (Skåne, Östergötland), transect (interior, edge), ploidy (diploid, tetraploid), field size, landscape proportion of arable land, flower density and presence of flower strip, and the interactions between flower strip presence and year, region, transect, and proportion arable land (including all 50 fields) or the size of the flower strip (including the 22 fields with flower strips), and the interaction between flower strip size and transect

	Bumble bee species		Bumble bees		Short‐tongued bumble bees		Long‐tongued bumble bees		Honey bees		CWM tongue length	
	*F*_df_	*p*	*F*_df_	*p*	*F*_df_	*p*	*F*_df_	*p*	*F*_df_	*p*	*F*_df_	*p*
All fields												
Year	0.04_1,43_	0.84	**39.17**_**1,35**_	**<0.0010**	**48.39**_**1,35**_	**<0.0010**	0.23_1,42_	0.63	3.87_1,34_	0.057	0.01_1,40_	0.94
Region	2.09_1,33_	0.16	**5.86**_**1,38**_	**0.021**	**6.35**_**1,37**_	**0.016**	0.03_1,42_	0.87	0.12_1,32_	0.73	0.03_1,38_	0.86
Transect	1.30_1,43_	0.26	0.56_1,44_	0.46	2.18_1,46_	0.15	**6.16**_**1,87**_	**0.015**	**5.01**_**1,39**_	**0.031**	**16.81**_**1,39**_	**<0.0010**
Ploidy	1.69_1,26_	0.20	1.71_1,36_	0.20	1.14_1,36_	0.29	0.25_1,42_	0.62	3.93_1,32_	0.056	2.35_1,39_	0.13
Field size	3.52_1,23_	0.074	3.65_1,36_	0.064	2.69_1,35_	0.11	3.90_1,51_	0.054	1.91_1,29_	0.18	4.09_1,34_	0.051
Arable	3.84_1,27_	0.060	0.84_1,35_	0.37	**4.46**_**1,35**_	**0.042**	0.21_1,41_	0.65	0.87_1,32_	0.36	0.90_1,37_	0.35
Flowers	0.23_1,50_	0.63	1.27_1,84_	0.26	2.87_1,87_	0.094	2.28_1,87_	0.13	0.99_1,74_	0.32	0.78_1,66_	0.38
Strip	**7.16**_**1,29**_	**0.012**	1.06_1,37_	0.31	1.09_1,39_	0.30	1.27_1,42_	0.27	0.23_1,33_	0.63	0.40_1,38_	0.53
Year × strip	**6.16**_**1,43**_	**0.017**	1.09_1,34_	0.30	0.38_1,34_	0.54	1.72_1,40_	0.20	3.15_1,33_	0.085	1.64_1,38_	0.21
Region × strip	0.26_1,28_	0.62	<0.01_1,35_	0.98	0.01_1,35_	0.94	0.18_1,44_	0.68	0.09_1,32_	0.77	0.04_1,37_	0.85
Transect × strip	2.68_1,44_	0.11	0.05_1,44_	0.83	0.12_1,46_	0.74	**5.81**_**1,87**_	**0.018**	0.45_1,40_	0.51	0.39_1,45_	0.54
Arable × strip	**4.64**_**1,28**_	**0.040**	0.49_1,36_	0.49	0.47_1,37_	0.50	0.97_1,42_	0.33	0.30_1,33_	0.59	0.24_1,37_	0.62
Fields with flower strip												
Year	2.46_1,14_	0.14	**92.01**_**1,15**_	**<0.0010**	**101.79**_**1,15**_	**<0.0010**	0.16_1,19_	0.69	3.14_1,11_	0.10	0.10_1,15_	0.75
Region	1.94_1,10_	0.19	1.19_1,19_	0.29	0.72_1,19_	0.41	<0.01_1,21_	0.96	0.14_1,9_	0.72	0.07_1,15_	0.80
Transect	0.32_1,14_	0.58	0.26_1,19_	0.62	0.44_1,19_	0.51	0.03_1,25_	0.85	1.05_1,18_	0.32	**6.38**_**1,20**_	**0.020**
Ploidy	<0.01_1,10_	0.96	0.06_1,19_	0.81	0.55_1,19_	0.47	0.20_1,21_	0.66	1.51_1,9_	0.25	1.16_1,15_	0.30
Field size	4.92_1,10_	0.051	**22.48**_**1,19**_	**<0.0010**	**14.20**_**1,19**_	**0.0013**	**6.90**_**1,25**_	**0.015**	0.08_1,10_	0.78	0.67_1,15_	0.40
Arable	4.23_1,10_	0.068	1.98_1,19_	0.18	**9.52**_**1,19**_	**0.0061**	0.27_1,16_	0.61	1.03_1,11_	0.33	0.31_1,16_	0.58
Flowers	<0.01_1,22_	0.96	2.22_1,19_	0.15	**4.44**_**1,19**_	**0.049**	1.11_1,34_	0.30	0.66_1,29_	0.42	0.01_1,32_	0.91
Strip size	0.01_1,15_	0.90	0.46_1,19_	0.51	0.46_1,19_	0.51	0.03_1,25_	0.86	1.52_1,12_	0.24	2.98_1,16_	0.10
Transect × strip	0.24_1,16_	0.63	<0.01_1,19_	0.95	0.01_1,19_	0.94	0.24_1,25_	0.63	2.10_1,18_	0.17	3.45_1,20_	0.078

*Note*

Bold numbers are for significant effects where *p* < 0.050. Least square mean values and the 95% confidence limits can be found in Supporting Information Table S3.

**Figure 4:**
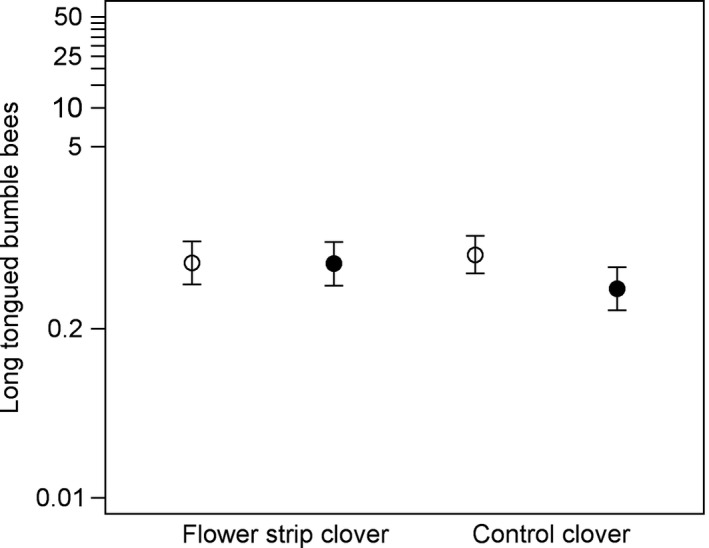
Density (bees per 50 m transect) of long‐tongued bumble bees in edge (open circles) and interior (filled circles) transects in clover fields with (*n* = 22) and without flower strip (control; *n* = 28). Means (circles) and 95% confidence limits (error bars) are based on model‐estimated least square means, back‐transformed using the ILINK option

Total bumble bee, short‐tongued bumble bee and honey bee densities over the whole season did not differ between clover fields with and without a flower strip or covary with the size of the flower strip (Table 1). Long‐tongued bumble bee density was explained by an interaction between flower strip presence and transect location, with lower density in the interior transect in clover fields without a flower strip (*F*_1,87_ = 11.43, *p* = 0.0011) but no difference between transects in clover fields with a flower strip (*F*_1,87_ < 0.01, *p* = 0.95) (Figure 4), and decreased with increasing field size (Table 1, Supporting Information Table S3). Total bumble bee density was higher in 2009 and in Skåne and decreased with increasing field size, but was not related to ploidy, arable land or flower density (Table 1, Supporting Information Table S3). Short‐tongued bumble bee density was, similar to total density, higher in 2009 and in Skåne and decreased with increasing field size, and also increased with proportion arable land in the landscape and flower density (Table 1, Supporting Information Table S3). Honey bee density was higher in edge transects, but was not significantly related to the other predictors (Table 1, Supporting Information Table S3). The CWM tongue length was not related to presence or size of flower strips, but related to transect location, with a longer tongued pollinator community in the interior transect (Table 1, Supporting Information Table S3).

**Figure 5:**
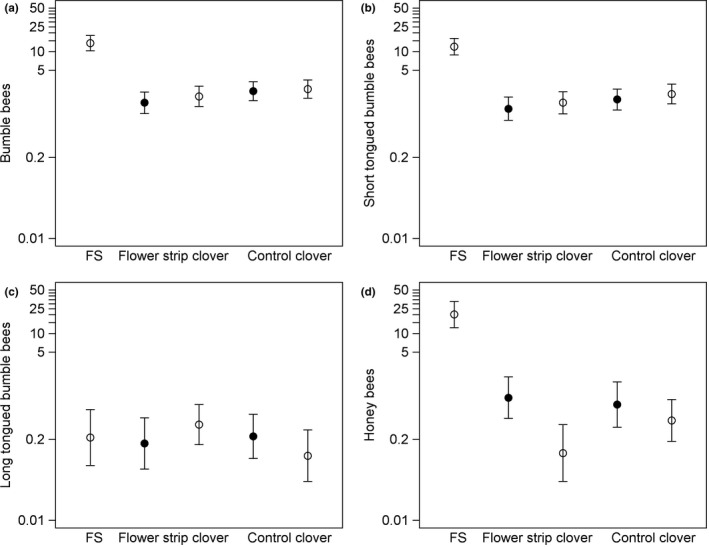
Densities (bees per 50 m transect) of (a) all bumble bees, (b) short‐tongued (<7 mm) and (c) long‐tongued (>7 mm) bumble bees and (d) honey bees, in transects in phacelia flower strips (FS) and in edge (filled circles) and interior (open circles) transects in clover fields with (*n* = 22) and without flower strip (control; *n* = 22) during the period when both phacelia and clover bloomed. Means (circles) and 95% confidence limits (error bars) are based on model‐estimated least square means, back‐transformed using the ILINK option

When both flower strips and clover fields flowered, both bumble bee and honey bee densities differed between transects (Table 2, Figure 5, Supporting Information Table S4). Densities of all bumble bees (*t*_1,13_ = 12.61, *p* < 0.0010) and short‐tongued bumble bees (*t*_1,13_ = 11.81, *p* < 0.0010) were higher in the phacelia than in the clover, but did not differ between edge and interior clover transects (all, *t*_1,57_ = 1.19, *p* = 0.24; short‐tongued, *t*_1,57_ = 1.01, *p* = 0.32). Density of long‐tongued bumble bees did not differ between transects (Table 2). Honey bee density was higher in the phacelia compared to the clover (*t*_1,27_ = 10.07, *p* < 0.0010) and was lower in the interior clover transect compared to the edge transect (*t*_1,34_ = 4.78, *p* < 0.0010). During the phacelia bloom period, total bumble bee and short‐tongued bumble bee densities did not differ between clover fields with and without a strip, while honey bee and long‐tongued bumble bee densities were explained by interactions between flower strip presence and transect location (Table 2). The density of long‐tongued bumble bees was higher in the interior transect in fields with a flower strip (*F*_1,34_ = 4.40, *p* = 0.044) but there was no difference in edge transects (*F*_1,36_ = 0.20, *p* = 0.66) (Figure 5c). Honey bee density showed a larger difference between transects in clover fields with a strip (*F*_1,58_ = 23.40, *p* < 0.0010) compared to without (*F*_1,74_ = 4.25, *p* = 0.043), with higher densities in edge compared to interior transects (Figure 5b).

**Table 2:** Numbers (bees per transect) of all bumble bees, short‐tongued (<7 mm) and long‐tongued (>7 mm) bumble bees and honey bees during the time period when both the phacelia and clover flowered, in relation to year (2009, 2010), region (Skåne, Östergötland), ploidy (diploid, tetraploid), field size, landscape proportion of arable land, flower density and, for fields with flower strip (*n* = 22), transect location (phacelia flower strip or adjacent edge or interior in the clover field) and, for all fields surveyed during the period (*n* = 44), transect location (edge or interior), and presence of flower strip

	Bumble bees		Short‐tongued bumble bees		Long‐tongued bumble bees		Honey bees	
	*F*_df_	*p*	*F*_df_	*p*	*F*_df_	*p*	*F*_df_	*p*
Fields with flower strip								
Year	**16.61**_**1,15**_	**<0.0010**	**14.77**_**1,16**_	**0.0014**	**5.53**_**1,14**_	**0.034**	**6.31**_**1,15**_	**0.024**
Region	4.34_1,15_	0.055	3.20_1,15_	0.094	2.04_1,30_	0.16	**7.50**_**1,17**_	**0.014**
Ploidy	0.15_1,15_	0.71	0.20_1,15_	0.66	1.31_1,20_	0.27	2.71_1,16_	0.12
Field size	0.32_1,15_	0.58	0.05_1,15_	0.83	1.21_1,18_	0.29	3.02_1,15_	0.10
Arable	<0.01_1,15_	0.97	0.01_1,15_	0.91	0.87_1,14_	0.37	4.23_1,14_	0.059
Flowers	**16.84**_**1,20**_	**<0.0010**	**15.44**_**1,20**_	**<0.0010**	1.28_1,16_	0.27	**4.11**_**1,40**_	**0.049**
Transect	**78.19**_**1,34**_	**<0.0010**	**68.52**_**1,33**_	**<0.0010**	1.02_1,26_	0.38	**50.23**_**1,31**_	**<0.0010**
All fields								
Year	**22.81**_**1,37**_	**<0.0010**	**20.80**_**1,45**_	**<0.0010**	**4.78**_**1,30**_	**0.037**	**9.04**_**1,49**_	**0.0042**
Region	**16.61**_**1,41**_	**<0.0010**	**10.78**_**1,44**_	**0.0020**	**5.63**_**1,29**_	**0.025**	3.98_1,42_	0.053
Ploidy	1.64_1,41_	0.21	1.26_1,30_	0.27	1.42_1,8_	0.21	0.20_1,29_	0.66
Field size	0.93_1,41_	0.34	3.14_1,52_	0.082	0.83_1,12_	0.38	1.44_1,46_	0.24
Arable	1.30_1,41_	0.26	0.71_1,41_	0.40	0.02_1,12_	0.89	0.04_1,42_	0.85
Flowers	**17.43**_**1,41**_	**<0.0010**	**20.38**_**1,78**_	**<0.0010**	0.04_1,12_	0.84	**12.40**_**1,74**_	**<0.0010**
Transect	2.44_1,41_	0.131	3.84_1,78_	0.0541	<0.01_1,78_	0.981	**28.40**_**1,61**_	**<0.0010**
Strip	2.15_1,41_	0.15	1.61_1,41_	0.21	0.95_1,14_	0.35	0.17_1,48_	0.68
Transect × strip	0.67_1,41_	0.42	0.01_1,78_	0.93	**4.15**_**1,78**_	**0.045**	**8.62**_**1,64**_	**0.0046**

*Note*

Bold numbers are for significant effects where *p* < 0.050. Least square mean values and the 95% confidence limits can be found in Supporting Information Table S4.

### 3.2 Pests and natural enemies

Weevil density was higher in 2009, in Skåne and in tetraploid clover, but was not related to presence or size of the flower strip (Table 3, Supporting Information Table S5). Parasitism rate of the natural enemies to the weevils was higher in 2010, in the edge transect and in fields with diploid clover and increased with flower density in fields with flower strips, but was not related to presence or size of the flower strip (Table 3, Supporting Information Table S5).

**Table 3:** *Protapion* spp. weevils per clover inflorescence, proportion of weevil larvae attacked by parasitoids and clover seed yield in relation to year (2009, 2010), region (Skåne, Östergötland), transect (interior, edge), ploidy (diploid, tetraploid), field size, landscape proportion of arable land, flower density, and presence of flower strip, and the interactions between flower strip presence and year, region, transect, and proportion arable land (including all 50 fields) or the size of the flower strip (including the 22 fields with flower strips) and the interaction between flower strip size and transect

	Weevils		Parasitism		Seed yield	
	*F*_df_	*p*	*F*_df_	*p*	*F*_df_	*p*
All fields						
Year	**15.98**_**1,35**_	**<0.0010**	**19.67**_**1,25**_	**<0.0010**	0.50_1,40_	0.48
Region	**6.81**_**1,39**_	**0.013**	1.46_1,44_	0.23	**13.68**_**1,40**_	**<0.0010**
Transect	0.23_1,48_	0.63	**21.82**_**1,54**_	**<0.0010**	2.01_1,47_	0.16
Ploidy	**7.28**_**1,38**_	**0.010**	**4.83**_**1,27**_	**0.037**	**11.77**_**1,39**_	**0.0014**
Field size	1.91_1,51_	0.17	2.52_1,34_	0.12	<0.01_1,39_	0.99
Arable	0.22_1,40_	0.64	0.01_1,28_	0.94	0.10_1,39_	0.75
Flowers	<0.01_1,56_	0.98	2.55_1,73_	0.11	2.35_1,70_	0.13
Strip	0.17_1,40_	0.68	0.69_1,31_	0.41	0.66_1,40_	0.42
Year × strip	2.63_1,35_	0.11	0.21_1,25_	0.65	1.77_1,40_	0.19
Region × strip	0.18_1,43_	0.67	0.96_1,44_	0.33	0.62_1,39_	0.44
Transect × strip	0.66_1,48_	0.42	0.61_1,51_	0.44	0.62_1,48_	0.43
Arable × strip	0.14_1,42_	0.71	1.57_1,33_	0.22	0.96_1,40_	0.33
Fields with flower strip						
Year	**4.91**_**1,12**_	**0.046**	**10.42**_**1,10**_	**0.0093**	0.99_1,14_	0.34
Region	**7.58**_**1,21**_	**0.012**	0.55_1,18_	0.47	**6.02**_**1,14**_	**0.028**
Transect	0.01_1,20_	0.91	**16.78**_**1,20**_	**<0.0010**	1.64_1,18_	0.22
Ploidy	0.77_1,18_	0.39	0.26_1,12_	0.62	**4.86**_**1,14**_	**0.045**
Field size	1.15_1,16_	0.30	1.47_1,6_	0.27	1.28_1,14_	0.28
Arable	0.55_1,16_	0.47	1.121_1,11_	0.97	2.01_1,14_	0.18
Flowers	1.96_1,23_	0.17	**4.56**_**1,25**_	**0.043**	**8.53**_**1,27**_	**0.0069**
Strip size	2.87_1,14_	0.11	0.90_1,8_	0.37	**8.79**_**1,15**_	**0.0098**
Transect × strip	0.52_1,20_	0.48	0.02_1,20_	0.88	0.08_1,18_	0.78

*Note*

Bold numbers are for significant effects where *p* < 0.050. Least square mean values and the 95% confidence limits can be found in Supporting Information Table S5.

### 3.3 Seed yield

**Figure 6:**
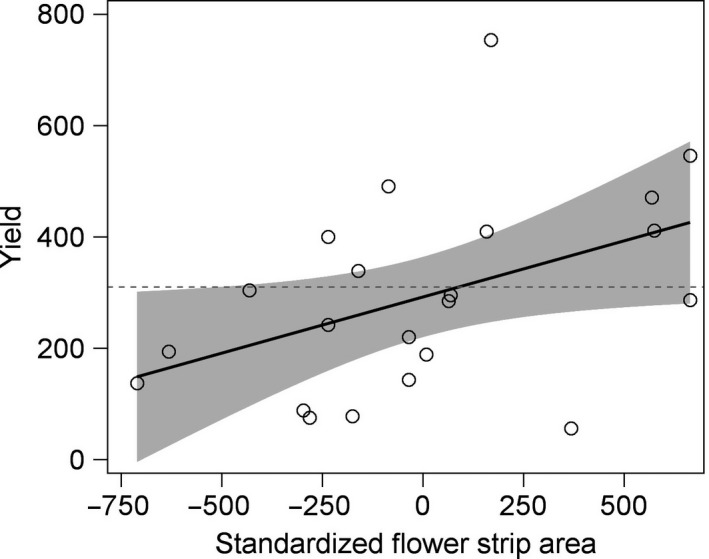
Clover seed yield (kg/ha) was positively related to phacelia flower strip area (standardized within clover cultivar ploidy level). The horizontal dashed line indicates the average seed yield in control fields and the shaded area shows 95% confidence limits for the linear relationship *n* = 22

**Figure 7:**
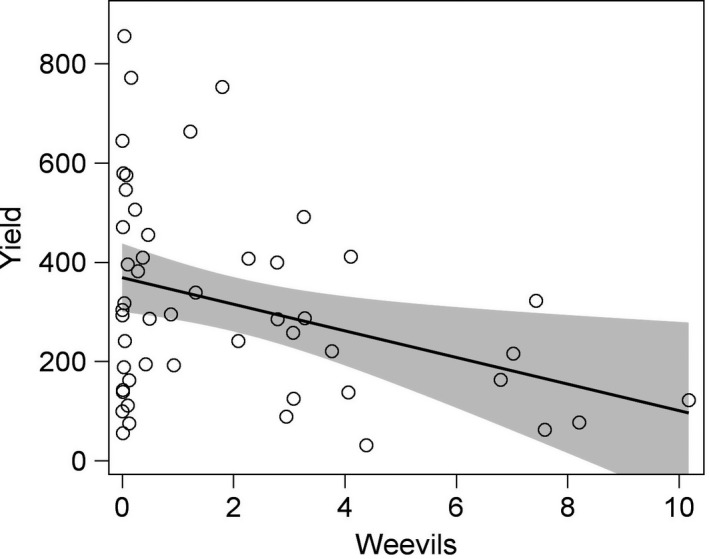
Clover seed yield (kg/ha) was negatively related to weevils per inflorescence. The shaded area indicates 95% confidence limits for the linear relationship *n* = 50

The clover seed yield was not related to the presence of flower strips, but increased with flower strip area (Table 3, Figure 6). Clover yields were higher in Skåne and for diploid clover and increased with flower density in fields with flower strips (Table 3, Supporting Information Table S5). Seed yield also decreased with increasing weevil density (*F*_1,44_ = 11.67, *p* = 0.0014; Figure 7), but was not related to bumble bee species richness (*F*_1,43_ = 0.15, *p* = 0.70) or density of short‐ (*F*_1,43_ = 0.05, *p* = 0.82) or long‐tongued bumble bees (*F*_1, 43_ = 0.21, *p* = 0.65) or honey bees (*F*_1,43_ = 2.42, *p* = 0.13).

## 4 DISCUSSION

Flower strips enhanced pollinator species richness and seed yield increased with strip area. Other than that, we found limited influence of the presence and size of flower strips on pollinators, biological control and yield. The strongest explanatory variables of seed yield were flower strip size, region, weevil infestation and ploidy and/or flower density, depending on model.

Interestingly, fields with flower strips harbored higher bumble bee species richness in the clover crop, with the strongest effect in more heterogeneous landscapes. A possibility is that a habitat enhancement in simplified landscapes can only draw species from a smaller pool of species (Kleijn et al., 2011). However, bumble bee species richness did not correlate with crop yield. Species richness has been established as a contributor to crop pollination services (Klein, Steffan‐Dewenter, & Tscharntke, 2003), but Swedish bumble bee communities are highly skewed in their species‐abundance distribution (Bommarco, Lundin, Smith, & Rundlöf, 2012). Species added to the community are often rare and are likely to contribute little to pollination (Kleijn et al., 2015; Winfree, Fox, Williams, Reilly, & Cariveau, 2015). Flower strips could still be important for conservation of less common bee species and insurance of ecosystem services by providing alternative and diverse forage resources. Bumble bee populations may also be limited by other factors than flower resource availability, such as nesting habitat (Öckinger & Smith, 2007).

When clover and phacelia co‐flowered, the phacelia was visited by higher densities of honey bees and bumble bees, the latter driven by short‐tongued bumble bee species. However, total bumble bee density in the clover did not change in fields with flower strips, either negatively during flower strip bloom or positively overall. The relatively small flower strip might add only limited resources for bumble bee population growth in the most common and short‐tongued bumble bee species and there could be enough other flowers available early in the season in southern Sweden. Mass‐flowering oilseed rape has been suggested to provide sufficient resources for growing large colonies of bumble bees in early summer, but not for later reproductive success (Persson & Smith, 2013; Westphal et al., 2009). We have found, for the Skåne region, that the late‐flowering red clover is itself an important resource for bumble bee population development across the season (Rundlöf et al., 2014). Bumble bee population persistence is thus rather the result of season‐long forage availability at a landscape scale (Carvell et al., 2017). Effects of flower strips on pollinators have also been observed to increase over years after establishment (Blaauw & Isaacs, 2014; Scheper et al., 2015), and this effect might have been lost for the monoculture annual flower strips we tested. In addition, more diverse flower strips could be more efficient than monocultures as they provide resources for a greater span of the season (Scheper et al., 2015).

The flower strips did, however, influence the distribution of honey bees and long‐tongued bumble bees when the phacelia and clover co‐flowered, with effects persisting throughout the season for the bumble bees. Flower strip presence contributed to increased density of long‐tongued bumble bees in the interior parts of the clover fields. This could be driven by competition with honey bees, because the density of honey bees was higher in clover field edges compared to interior parts when phacelia and clover co‐flowered and this difference was much larger in fields with a flower strip. Our interpretation is that the flower strips redistribute honey bees from the interior parts of clover fields to the clover field edge adjacent to the phacelia. Honey bees could be visiting the more easily accessible phacelia mainly for nectar and use the adjacent clover for pollen collection, possibly with some bees collecting nectar and pollen from the two different plant species on the same or consecutive foraging bouts (Free, 1963). Decreasing reward levels could reduce flower constancy (Grüter, Moore, Firmin, Helanterä, & Ratnieks, 2011) and result in bees visiting both plant species. Our results point to phacelia flower strip needing to be cut once the clover blooms to avoid reduced honey bee density in the interior parts of clover fields. However, keeping the phacelia flowers uncut could be an option to support a more even distribution of long‐tongued bumble bees throughout the clover fields.

We found no relationships between either the presence or size of flower strips and pest densities or parasitism rates, indicating that phacelia does not provide resources or shelter to neither weevils nor their parasitoids. Parasitic wasps can benefit from flower plantings near the crop, providing them with nectar, pollen and shelter (Jonsson et al., 2010), but we have no information on the potential benefit of phacelia for the most common parasitoids of the weevil pests. Parasitism was instead strongly related to transect location, with higher parasitism rates in edge transects compared with interior transects, despite similar weevil densities in both transects. This indicates that the parasitoids have a limited dispersal range. Parasitism rates varied considerably among fields and understanding the population biology and resource needs of the parasitic wasps is needed to design measures to support them (Shaw & Hochberg, 2001). Weevil infestation was strongly related to seed yield, and developing pest control measures emerges as a priority. It would be interesting to identify other natural enemies to the weevil pest, such as generalist arthropod predators (Roubinet et al., 2017), and examine how establishment of resource providing plants influence them in clover.

Addition of an early blooming flower resource, such as phacelia grown at red clover seed fields, can support additional bumble bee species, particularly in more heterogeneous landscapes. Future studies could focus on finding plant mixes that would benefit both crop pollinators and a diverse pollinator community, to increase the benefits further (Williams & Lonsdorf, 2018). Fields with added flower resources had, however, similar levels of pollinator density and biological control of the pests as control fields. The ecology underpinning the positive relationship between flower strip size and clover seed yield deserves more scrutiny, with ploidy level taken into account. The relationship could possibly have emerged from the combination of nonsignificantly positive relationships between strip area and bee densities, and a nonsignificantly negative relationship between strip area and weevil density. Irrespective of mechanism, the result suggests that flower strips, if sufficiently large, could be an option to increased crop yield, but this needs to be investigated in future studies that consistently include larger flower strips. The clover seed yield was mainly limited by weevil infestation, and a future focus should be to design targeted support measures for pest control services.

## CONFLICT OF INTEREST

The authors declare no conflict of interest.

## AUTHOR CONTRIBUTIONS

All authors conceived the ideas and designed methodology; MR and OL collected data; MR analyzed the data and led the writing of the manuscript. All authors contributed to the interpretation of results and writing.

## DATA ACCESSIBILITY

Data is available via the Dryad Digital Repository. doi:10.5061/dryad.1h5717p

## References

[ece34330-bib-0001] Bianchi, F. J. J. A. , Ives, A. R. , & Schellhorn, N. A. (2013). Interactions between conventional and organic farming for biocontrol services across the landscape. Ecological Applications, 23, 1531–1543. 10.1890/12-1819.1 24261038

[ece34330-bib-0002] Blaauw, B. R. , & Isaacs, R. (2014). Flower plantings increase wild bee abundance and the pollination services provided to a pollination‐dependent crop. Journal of Applied Ecology, 51, 890–898. 10.1111/1365-2664.12257

[ece34330-bib-0003] Bommarco, R. , & Fagan, W. F. (2002). Influence of crop edges on movement of generalist predators: A diffusion approach. Agricultural and Forest Entomology, 4, 21–30. 10.1046/j.1461-9563.2002.00117.x

[ece34330-bib-0004] Bommarco, R. , Kleijn, D. , & Potts, S. G. (2013). Ecological intensification: Harnessing ecosystem services for food security. Trends in Ecology & Evolution, 28, 230–238. 10.1016/j.tree.2012.10.012 23153724

[ece34330-bib-0005] Bommarco, R. , Lundin, O. , Smith, H. G. , & Rundlöf, M. (2012). Drastic historic shifts in bumble‐bee community composition in Sweden. Proceedings of the Royal Society B: Biological Sciences, 279, 309–315. 10.1098/rspb.2011.0647 21676979 PMC3223670

[ece34330-bib-0006] Carvalheiro, L. G. , Seymour, C. L. , Nicolson, S. W. , & Veldtman, R. (2012). Creating patches of native flowers facilitates crop pollination in large agricultural fields: Mango as a case study. Journal of Applied Ecology, 49, 1373–1383. 10.1111/j.1365-2664.2012.02217.x

[ece34330-bib-0007] Carvell, C. , Bourke, A. F. G. , Dreier, S. , Freeman, S. N. , Hulmes, S. , Jordan, W. C. , … Heard, M. S. (2017). Bumblebee family lineage survival is enhanced in high‐quality landscapes. Nature, 543, 547–549. 10.1038/nature21709 28297711

[ece34330-bib-0008] Carvell, C. , Osborne, J. L. , Bourke, A. F. G. , Freeman, S. N. , Pywell, R. F. , & Heard, M. S. (2011). Bumble bee species’ responses to a targeted conservation measure depend on landscape context and habitat quality. Ecological Applications, 21, 1760–1771. 10.1890/10-0677.1 21830716

[ece34330-bib-0009] Díaz, S. , Lavorel, S. , de Bello, F. , Quétier, F. , Grigulis, K. , & Robson, T. M. (2007). Incorporating plant functional diversity effects in ecosystem service assessments. Proceedings of the National Academy of Sciences, 104, 20684–20689. 10.1073/pnas.0704716104 PMC2410063 18093933

[ece34330-bib-0010] Edwards, E. , & Jenner, M. (2005). Field guide to the bumblebees of Great Britain and Ireland. Eastbourne, UK: Ocelli Limited.

[ece34330-bib-0011] Free, J. B. (1963). The flower constancy of honeybees. Journal of Animal Ecology, 32, 119–131. 10.2307/2521

[ece34330-bib-0012] Free, J. B. (1993). Insect pollination of crops. London, UK: Academic Press.

[ece34330-bib-0013] Garibaldi, L. A. , Bartomeus, I. , Bommarco, R. , Klein, A. M. , Cunningham, S. A. , Aizen, M. A. , … Woyciechowski, M. (2015). Trait matching of flower visitors and crops predicts fruit set better than trait diversity. Journal of Applied Ecology, 52, 1436–1444. 10.1111/1365-2664.12530

[ece34330-bib-0014] Garibaldi, L. A. , Carvalheiro, L. G. , Leonhardt, S. D. , Aizen, M. A. , Blaauw, B. R. , Isaacs, R. , … Winfree, R. (2014). From research to action: Enhancing crop yield through wild pollinators. Frontiers in Ecology and the Environment, 12, 439–447. 10.1890/130330

[ece34330-bib-0015] Garibaldi, L. A. , Steffan‐Dewenter, I. , Kremen, C. , Morales, J. M. , Bommarco, R. , Cunningham, S. A. , … Klein, A. M. (2011). Stability of pollination services decreases with isolation from natural areas despite honey bee visits. Ecology Letters, 14, 1062–1072. 10.1111/j.1461-0248.2011.01669.x 21806746

[ece34330-bib-0016] Grüter, C. , Moore, H. , Firmin, N. , Helanterä, H. , & Ratnieks, F. L. W. (2011). Flower constancy in honey bee workers (*Apis mellifera*) depends on ecologically realistic rewards. The Journal of Experimental Biology, 214, 1397–1402. 10.1242/jeb.050583 21430217

[ece34330-bib-0017] Holzschuh, A. , Dormann, C. F. , Tscharntke, T. , & Steffan‐Dewenter, I. (2011). Expansion of mass‐flowering crops leads to transient pollinator dilution and reduced wild plant pollination. Proceedings of the Royal Society B: Biological Sciences, 278, 3444–3451. 10.1098/rspb.2011.0268 21471115 PMC3177631

[ece34330-bib-0018] Jonsson, M. , Wratten, S. D. , Landis, D. A. , Tompkins, J. M. , & Cullen, R. (2010). Habitat manipulation to mitigate the impacts of invasive arthropod pests. Biological Invasions, 12, 2933–2945. 10.1007/s10530-010-9737-4

[ece34330-bib-0019] Kennedy, C. M. , Lonsdorf, E. , Neel, M. C. , Williams, N. M. , Ricketts, T. H. , Winfree, R. , … Kremen, C. (2013). A global quantitative synthesis of local and landscape effects on wild bee pollinators in agroecosystems. Ecology Letters, 16, 584–599. 10.1111/ele.12082 23489285

[ece34330-bib-0020] Kleijn, D. , Rundlöf, M. , Scheper, J. , Smith, H. G. , & Tscharntke, T. (2011). Does conservation on farmland contribute to halting the biodiversity decline? Trends in Ecology & Evolution, 26, 474–481. 10.1016/j.tree.2011.05.009 21703714

[ece34330-bib-0021] Kleijn, D. , Winfree, R. , Bartomeus, I. , Carvalheiro, L. G. , Henry, M. , Isaacs, R. , … Potts, S. G. (2015). Delivery of crop pollination services is an insufficient argument for wild pollinator conservation. Nature Communications, 6, 7414 10.1038/ncomms8414 PMC4490361 26079893

[ece34330-bib-0022] Klein, A. M. , Steffan‐Dewenter, I. , & Tscharntke, T. (2003). Fruit set of highland coffee increases with the diversity of pollinating bees. Proceedings of the Royal Society B: Biological Sciences, 270, 955–961. 10.1098/rspb.2002.2306 12803911 PMC1691323

[ece34330-bib-0023] Klein, A. M. , Vaissiere, B. E. , Cane, J. H. , Steffan‐Dewenter, I. , Cunningham, S. A. , Kremen, C. , & Tscharntke, T. (2007). Importance of pollinators in changing landscapes for world crops. Proceedings of the Royal Society B: Biological Sciences, 274, 303–313. 10.1098/rspb.2006.3721 17164193 PMC1702377

[ece34330-bib-0024] Kruess, A. , & Tscharntke, T. (1994). Habitat fragmentation, species loss, and biological‐control. Science, 264, 1581–1584. 10.1126/science.264.5165.1581 17769603

[ece34330-bib-0025] Littell, R. C. , Milliken, G. A. , Stroup, W. W. , Wolfinger, R. D. , & Schabenberger, O. (2006). SAS for mixed models (2nd ed.). Cary, NC: SAS Institute Inc..

[ece34330-bib-0026] Løken, A. (1973). Studies on Scandinavian bumble bees (Hymenoptera, Apidae). Norwegian Journal of Entomology, 20, 1–218.

[ece34330-bib-0027] Losey, J. E. , & Vaughan, M. (2006). The economic value of ecological services provided by insects. BioScience, 56, 311–323. 10.1641/0006-3568(2006)56[311:TEVOES]2.0.CO;2

[ece34330-bib-0028] Lundin, O. , Rundlöf, M. , Smith, H. G. , & Bommarco, R. (2012). Towards integrated pest management in red clover seed production. Journal of Economic Entomology, 105, 1620–1628. 10.1603/EC12179 23156158

[ece34330-bib-0029] Lundin, O. , Ward, K. L. , Artz, D. R. , Boyle, N. K. , Pitts‐Singer, T. L. , & Williams, N. M. (2017). Wildflower plantings do not compete with neighboring almond orchards for pollinator visits. Environmental Entomology, 46, 559–564. 10.1093/ee/nvx052 28379320

[ece34330-bib-0030] Öckinger, E. , & Smith, H. G. (2007). Semi‐natural grasslands as population sources for pollinating insects in agricultural landscapes. Journal of Applied Ecology, 44, 50–59.

[ece34330-bib-0031] Persson, A. S. , & Smith, H. G. (2013). Seasonal persistence of bumblebee populations is affected by landscape context. Agriculture, Ecosystems & Environment, 165, 201–209. 10.1016/j.agee.2012.12.008

[ece34330-bib-0032] Polis, G. A. , Anderson, W. B. , & Holt, R. D. (1997). Towards an integration of landscape and food web ecology: The dynamics of spatially subsidized food webs. Annual Review of Ecology and Systematics, 28, 289–316. 10.1146/annurev.ecolsys.28.1.289

[ece34330-bib-0033] Prys‐Jones, O. E. , & Corbet, S. A. (1986). Bumblebees. Cambridge, UK: Cambridge University Press.

[ece34330-bib-0034] Rand, T. A. , Tylianakis, J. M. , & Tscharntke, T. (2006). Spillover edge effects: The dispersal of agriculturally subsidized insect natural enemies into adjacent natural habitats. Ecology Letters, 9, 603–614. 10.1111/j.1461-0248.2006.00911.x 16643305

[ece34330-bib-0035] Riedinger, V. , Renner, M. , Rundlöf, M. , Steffan‐Dewenter, I. , & Holzschuh, A. (2014). Early mass‐flowering crops mitigate pollinator dilution in late‐flowering crops. Landscape Ecology, 29, 425–435. 10.1007/s10980-013-9973-y

[ece34330-bib-0036] Roubinet, E. , Birkhofer, K. , Malsher, G. , Staudacher, K. , Ekbom, B. , Traugott, M. , & Jonsson, M. (2017). Diet of generalist predators reflects effects of cropping period and farming system on extra‐ and intraguild prey. Ecological Applications, 27, 1167–1177. 10.1002/eap.1510 28132400

[ece34330-bib-0037] Rundlöf, M. , Persson, A. S. , Smith, H. G. , & Bommarco, R. (2014). Late‐season mass‐flowering red clover increases bumble bee queen and male densities. Biological Conservation, 172, 138–145. 10.1016/j.biocon.2014.02.027

[ece34330-bib-0038] Rusch, A. , Chaplin‐Kramer, R. , Gardiner, M. M. , Hawro, V. , Holland, J. , Landis, D. , … Bommarco, R. (2016). Agricultural landscape simplification reduces natural pest control: A quantitative synthesis. Agriculture, Ecosystems & Environment, 221, 198–204. 10.1016/j.agee.2016.01.039

[ece34330-bib-0039] Schellhorn, N. A. , Parry, H. R. , Macfadyen, S. , Wang, Y. M. , & Zalucki, M. P. (2015). Connecting scales: Achieving in‐field pest control from areawide and landscape ecology studies. Insect Science, 22, 35–51. 10.1111/1744-7917.12161 25099692

[ece34330-bib-0040] Scheper, J. , Bommarco, R. , Holzschuh, A. , Potts, S. G. , Riedinger, V. , Roberts, S. P. M. , … Kleijn, D. (2015). Local and landscape‐level floral resources explain effects of wildflower strips on wild bees across four European countries. Journal of Applied Ecology, 52, 1165–1175. 10.1111/1365-2664.12479

[ece34330-bib-0041] Shaw, M. R. , & Hochberg, M. E. (2001). The neglect of parasitic Hymenoptera in insect conservation strategies: The British fauna as a prime example. Journal of Insect Conservation, 5, 253–263. 10.1023/A:1013393229923

[ece34330-bib-0042] Smith, H. G. , Birkhofer, K. , Clough, Y. , Ekroos, J. , Olsson, O. , & Rundlöf, M. (2014). Beyond dispersal: The role of animal movement in modern agricultural landscapes In Hansson L.‐A. & Kesson S. (Eds.), Animal movement across scales (pp. 62–87). Oxford, UK: Oxford University Press.

[ece34330-bib-0043] Tilman, D. , Balzer, C. , Hill, J. , & Befort, B. L. (2011). Global food demand and the sustainable intensification of agriculture. Proceedings of the National Academy of Sciences, 108, 2060–2064. 10.1073/pnas.1116437108 PMC3250154 22106295

[ece34330-bib-0044] Tschumi, M. , Albrecht, M. , Entling, M. H. , & Jacot, K. (2015). High effectiveness of tailored flower strips in reducing pests and crop plant damage. Proceedings of the Royal Society B: Biological Sciences, 282, 20151369 10.1098/rspb.2015.1369 PMC4571701 26311668

[ece34330-bib-0045] van Rijn, P. C. J. , & Wäckers, F. L. (2016). Nectar accessibility determines fitness, flower choice and abundance of hoverflies that provide natural pest control. Journal of Applied Ecology, 53, 925–933. 10.1111/1365-2664.12605

[ece34330-bib-0046] Vleugels, T. , Roldan‐Ruiz, I. , & Cnops, G. (2015). Influence of flower and flowering characteristics on seed yield in diploid and tetraploid red clover. Plant Breeding, 134, 56–61. 10.1111/pbr.12224

[ece34330-bib-0047] Westphal, C. , Steffan‐Dewenter, I. , & Tscharntke, T. (2003). Mass flowering crops enhance pollinator densities at a landscape scale. Ecology Letters, 6, 961–965. 10.1046/j.1461-0248.2003.00523.x

[ece34330-bib-0048] Westphal, C. , Steffan‐Dewenter, I. , & Tscharntke, T. (2009). Mass flowering oilseed rape improves early colony growth but not sexual reproduction of bumblebees. Journal of Applied Ecology, 46, 187–193. 10.1111/j.1365-2664.2008.01580.x

[ece34330-bib-0049] Williams, N. M. , & Lonsdorf, E. V. (2018). Selecting cost‐effective plant mixes to support pollinators. Biological Conservation, 217, 195–202. 10.1016/j.biocon.2017.10.032

[ece34330-bib-0050] Williams, N. M. , Regetz, J. , & Kremen, C. (2012). Landscape‐scale resources promote colony growth but not reproductive performance of bumble bees. Ecology, 93, 1049–1058. 10.1890/11-1006.1 22764491

[ece34330-bib-0051] Winfree, R. , Fox, J. W. , Williams, N. M. , Reilly, J. R. , & Cariveau, D. P. (2015). Abundance of common species, not species richness, drives delivery of real‐world ecosystem services. Ecology Letters, 18, 626–635. 10.1111/ele.12424 25959973

[ece34330-bib-0052] Zuur, A. F. , Ieno, E. N. , & Elphick, C. S. (2010). A protocol for data exploration to avoid common statistical problems. Methods in Ecology and Evolution, 1, 3–14. 10.1111/j.2041-210X.2009.00001.x

