# Supplementary figures and images for: Annual flower strips support pollinators and potentially enhance red clover seed yield
Source: Ecol Evol. 2018 Jul 16;8(16):7974–85. doi: 10.1002/ece3.4330 (PMC6144972; doi:10.1002/ece3.4330)

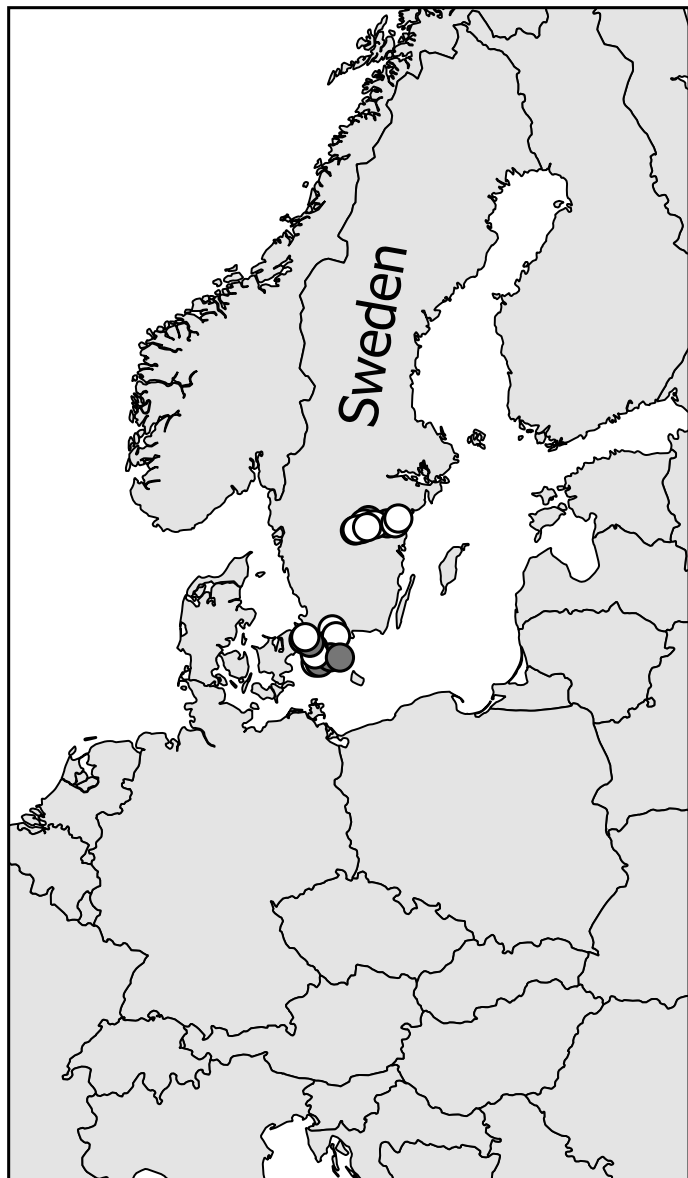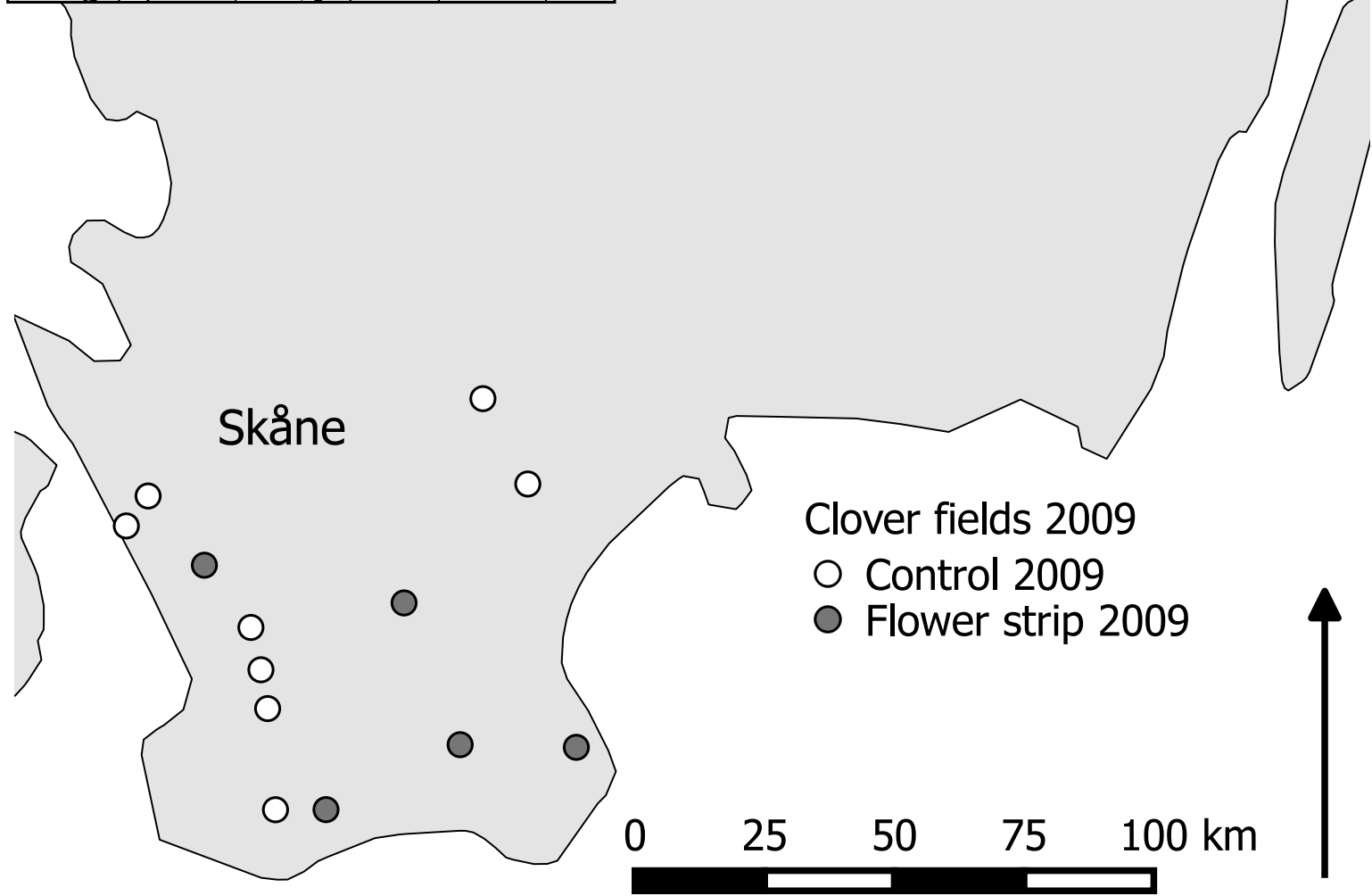

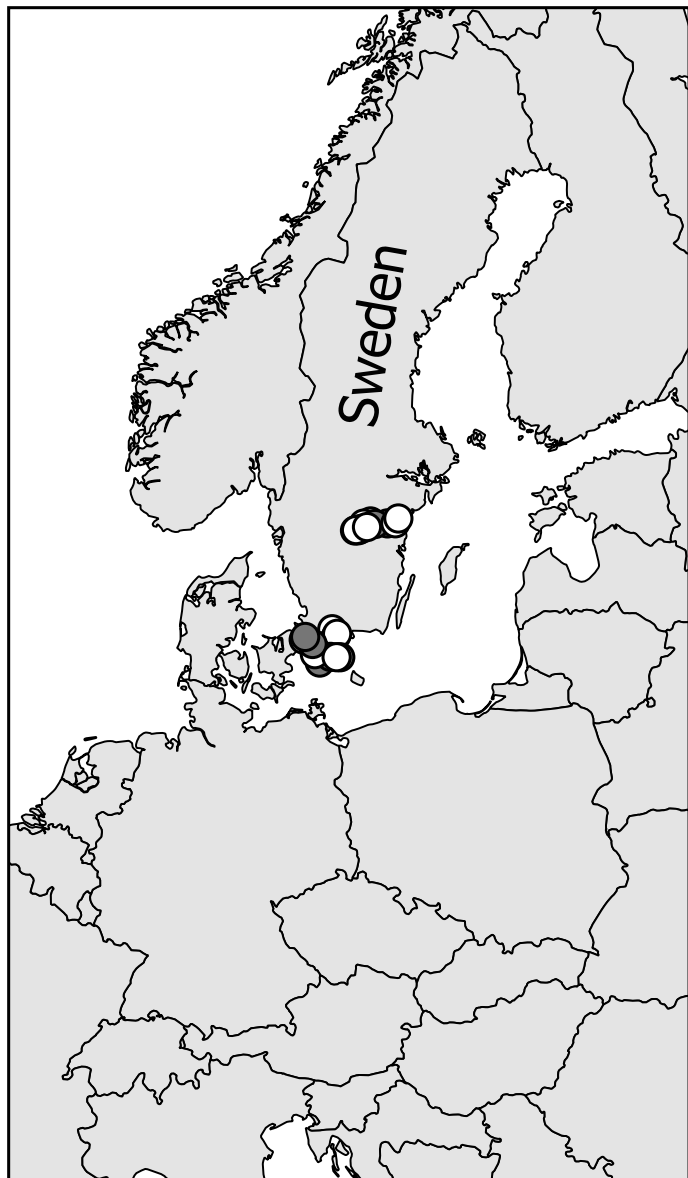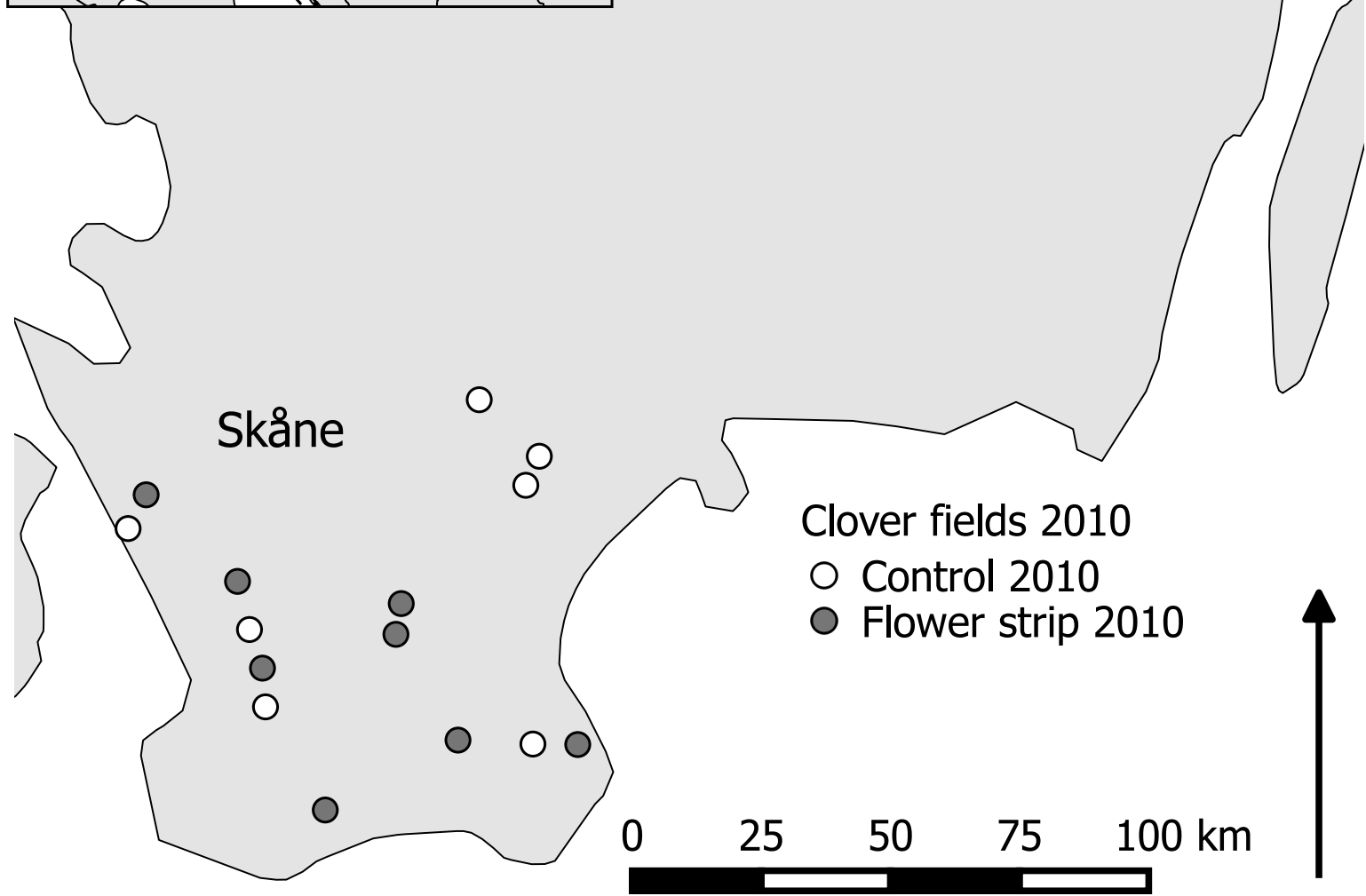

Supplement: Supplementary file 1 [file ECE3-8-7974-s001.pdf]
