# Supplementary material for: Annual flower strips support pollinators and potentially enhance red clover seed yield
Source: Ecol Evol. 2018 Jul 16;8(16):7974–85. doi: 10.1002/ece3.4330 (PMC6144972; doi:10.1002/ece3.4330)
Supplement: Supplementary file 2 [file ECE3-8-7974-s002.pdf]

Supplementary Information for

Annual flower strips support pollinators and potentially enhance red clover seed yield

Maj Rundlöf, Ola Lundin & Riccardo Bommarco

Correspondence to: maj.rundlof@biol.lu.se

This PDF file includes:

Materials and methods

Fig. S1

Tables S1-S5

Materials and methods

FLOWER STRIP ESTABLISHMENT

Strips of phacelia *Phacelia tanacetafolia* were sown on the edge in 9 of the 24 fields in 2009 (5 in Skåne and 4 in Östergötland) and in 13 of the 26 fields in 2010 (8 in Skåne and 5 in Östergötland) (Fig. S1). We chose phacelia because it is easy to establish and has been used successfully to create pollinator habitat on arable land (e.g. Pywell *et al.* 2005) and because we predicted that the relatively open flowers would be attractive to the major crop pollinators (Kleijn *et al.* 2015). The strips were sown from the middle to the end of April, with a seeding density of 10 kg phacelia seeds ha^{-1}. The phacelia started to flower around the middle of June. Surveys in phacelia flower strips and clover fields, when both were in bloom, were conducted 30 June-16 July 2009 and 6-12 July 2010 in Skåne and 2-7 July 2009 and 8-9 July 2010 in Östergötland. The flower strip area ranged from 538 to 1600 m^2 (mean 1198 m^2) in 2009 and from 125 to 2000 m^2 (997 m^2) in 2010 (Table S1). Phacelia plant density was counted in four 0.5×0.5 m squares with equal spacing along a 50 m transect in the flower strip once the strip had started flowering. The average number of phacelia plants was 97 plants m^{-2} (range: 44-148) in 2009 and 132 plants m^{-2} (57-317) in 2010.

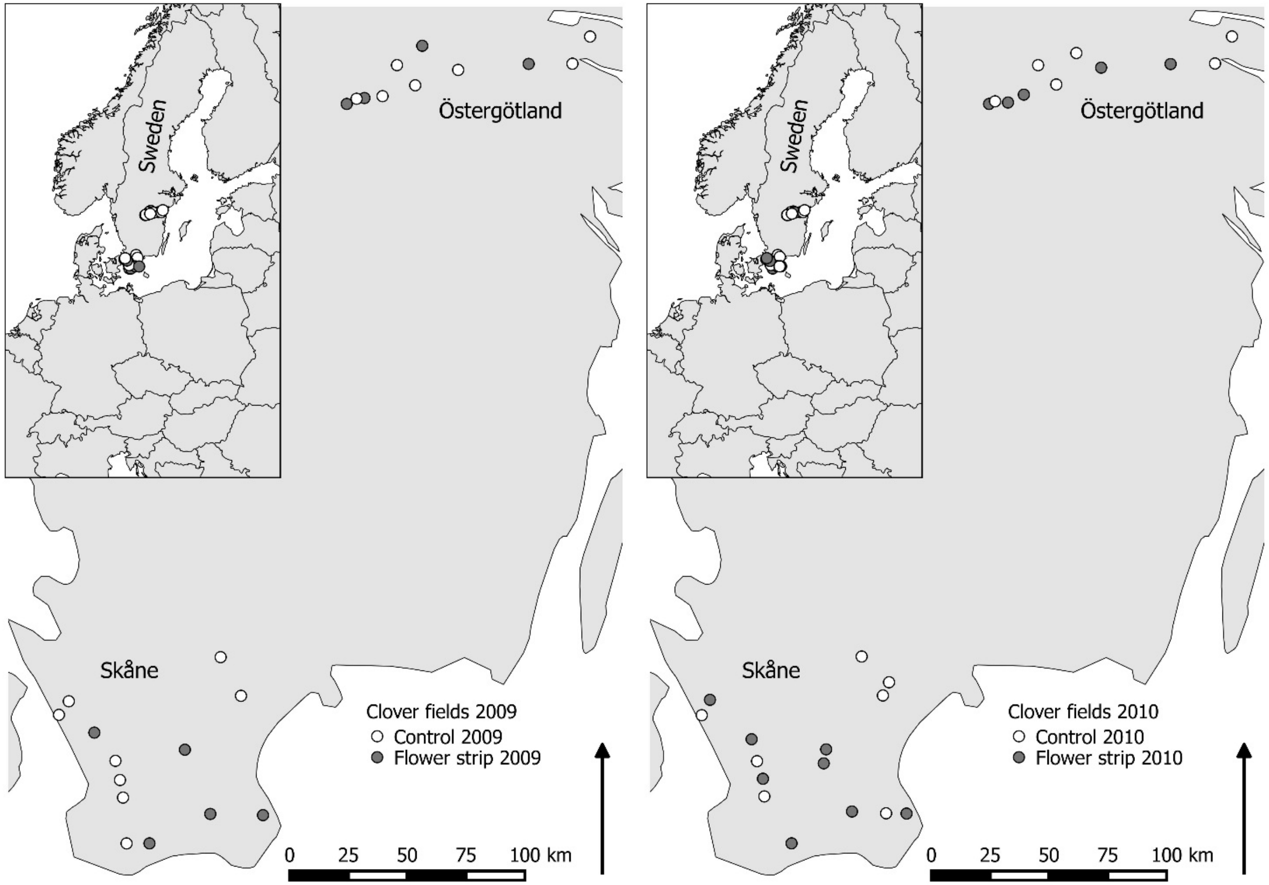

Fig. S1. Red clover seed fields with or without (control) a phacelia flower strip in the two study regions (Skåne and Östergötland) in Sweden over the two years of study (2009-2010).

Table S1. Number of red clover *Trifolium pratense* fields included in the study over the two years with and without sown strips of phacelia *Phacelia tanacetifolia*, ploidy of the clover cultivar, size of clover fields and sown flower strips, proportion of arable land and semi-natural grassland in the 1 km radius landscapes surrounding the clover fields and number of honey bee colonies per hectare clover field in 2010 (no data available for 2009). There were no significant differences in clover field size (Wilcoxon test, Z = 0.94, P = 0.35), proportion of arable land (PROC GLM, $F_{1, 48}$ = 1.07, P = 0.31) or proportion of semi-natural grassland (Z = 0.79, P = 0.43) in the landscape or honey bee colonies per hectare (Z = 1.82, P = 0.069) between fields with and without flower strips.

			Ploidy		Clover field size (ha)			Flower strip area (m^2)			Proportion arable land (%)			Proportion semi-natural grassland (%)			Honey bee colonies per ha clover field		
Year	Flower strip	N	2n	4n	Min	Mean	Max	Min	Mean	Max	Min	Mean	Max	Min	Mean	Max	Min	Mean	Max
2009	No	15	9	6	4.0	9.7	24				28	54	76	0.99	4.7	13			
	Yes	9	4	5	6.0	8.6	15	538	1198	1600	17	52	76	1.7	5.6	12			
2010	No	13	7	6	6.5	9.5	21				22	52	71	1.2	5.7	15	0	0.70	3.1
	Yes	13	5	8	5.0	8.5	15	125	997	2000	14	46	74	1.2	6.4	18	0	1.3	3.8
All fields		50	25	25		9.1			1058			51			5.6				

Table S2. Bee abundances in the clover fields and tongue lengths and tongue length classes (short, <7 mm; long >7 mm) for the non-parasitic bumble bee species in the study and the literature sources for the trait data. *Bombus terrestris*, *Bombus lucorum*, *Bombus magnus* and *Bombus cryptarum* were grouped (*Bombus terrestris* aggr.) due to difficulties in separating these species morphologically (Murray *et al.* 2008).

Species	N individuals	Tongue length (mm)	Bumble bee tongue length class	Source
Apis mellifera	2723	4.98		Tongue length measured on 30 workers collected in the region (at the same time as the material and following the methods in Persson *et al*. (2015)).
Bombus distinguendus	23	9.70	long	Goulson *et al*. (2005)
Bombus hortorum	80	10.55	long	Persson *et al*. (2015)
Bombus lapidarius	483	6.07	short	Persson *et al*. (2015)
Bombus muscorum	11	8.80	long	Goulson *et al*. (2005)
Bombus pascuorum	50	7.13	long	Persson *et al*. (2015)
Bombus pratorum	2	5.91	short	Persson *et al*. (2015)
Bombus ruderarius	10	6.90	short	Persson *et al*. (2015)
Bombus soroeensis	51	5.62	short	Persson *et al*. (2015)
Bombus subterraneus	112	8.60	long	Persson *et al*. (2015)
Bombus sylvarum	42	6.84	short	Persson *et al*. (2015)
Bombus terrestris aggr.	3021	6.99	short	Persson *et al*. (2015)
Bombus campestris	1			
Bombus rupestris	5			
Bombus spp.	148			

Table S3. Bumble bee species (per transect over the season), densities (bees per 50 m transect and survey round) of all bumble bees and short tongued (<7 mm) and long tongued (>7 mm) bumble bees and honey bees and community weighted mean (CWM) bee tongue length in the clover fields for the different years, regions, transects and ploidy (2n, diploid; 4n, tetraploid), in relation to the proportion of arable land in the 1 km circular landscape surrounding the clover field and presence of flower strip (including all 50 fields) or size of the flower strip (m^2) (including the 22 fields with flower strips). Numbers indicate least square mean values and the 95% confidence limits (cl) estimated from the statistical models, back-transformed using the ILINK option when needed, and the model-estimated slope for the continuous variables field size, arable land, flower density and flower strip area. Bold numbers are for significant effects where P <0.050 (see Table 1).

		Bumble bee species estimate (cl)	Bumble bees estimate (cl)	Short tongued bumble bees estimate (cl)	Long tongued bumble bees estimate (cl)	Honey bees estimate (cl)	CWM tongue length estimate (cl)
All fields							
Year	2009	5.0 (4.6-5.4)	**13 (11-16)**	**11 (9.6-13)**	0.56 (0.40-0.78)	7.1 (4.7-11)	6.25 (6.06-6.43)
	2010	4.9 (4.4-5.4)	**6.6 (5.6-7.8)**	**4.8 (4.0-5.8)**	0.62 (0.45-0.86)	4.1 (2.9-6.0)	6.24 (6.06-6.41)
Region	Skåne	4.7 (4.2-5.1)	**11 (9.4-13)**	**8.9 (7.4-11)**	0.59 (0.42-0.80)	5.7 (3.9-8.4)	6.23 (6.05-6.40)
	Östergötland	5.2 (4.7-5.7)	**8.1 (6.7-9.7)**	**6.3 (5.1-7.6)**	0.60 (0.42-0.87)	5.2 (3.3-7.9)	6.25 (6.06-6.45)
Transect	interior	4.8 (4.4-5.2)	9.6 (8.5-11)	7.7 (6.8-8.8)	**0.51 (0.39-0.66)**	**4.8 (3.6-6.5)**	**6.34 (6.20-6.47)**
	edge	5.1 (4.7-5.5)	9.3 (8.2-10)	7.2 (6.3-8.2)	**0.69 (0.54-0.89)**	**6.1 (4.5-8.1)**	**6.15 (6.01-6.28)**
Ploidy	2n	5.2 (4.7-5.6)	10 (8.7-12)	8.0 (6.7-9.6)	0.63 (0.45-0.88)	7.3 (4.9-11)	6.14 (5.96-6.31)
	4n	4.7 (4.2-5.2)	8.7 (7.2-10)	6.9 (5.7-8.4)	0.55 (0.39-0.80)	4.0 (2.6-8.1)	6.35 (6.15-6.55)
Field size		-0.084	-0.032	-0.030	-0.069	0.053	-0.036
Arable		-4.3	0.072	**0.56**	-1.1	-1.3	0.17
Flowers		-0.0064	0.0039	0.0067	-0.012	0.0073	-0.0031
Strip	no	**4.6 (4.2-5.0)**	8.7 (7.5-10)	6.8 (5.7-8.1)	0.55 (0.40-0.75)	5.5 (3.8-8.0)	6.21 (6.04-6.37)
	yes	**5.3 (4.8-5.9)**	10 (8.5-12)	8.1 (6.7-9.8)	0.64 (0.45-0.90)	5.3 (3.5-8.1)	6.28 (6.09-6.47)
Fields with flower strip							
Year	2009	5.9 (5.1-6.7)	**15 (14-17)**	**13 (11-15)**	0.72 (0.42-1.2)	7.5 (3.8-15)	6.27 (5.98-6.56)
	2010	5.1 (4.3-5.9)	**6.9 (6.0-7.9)**	**5.1 (4.4-5.9)**	0.63 (0.39-1.0)	3.6 (2.0-6.5)	6.33 (6.08-6.57)
Region	Skåne	5.0 (4.1-5.9)	11 (9.5-13)	8.5 (7.2-10)	0.66 (0.37-1.2)	5.7 (2.8-12)	6.27 (5.97-6.57)
	Östergötland	6.0 (5.0-6.9)	9.7 (8.3-11)	7.7 (6.5-9.1)	0.68 (0.37-1.3)	4.7 (2.2-10)	6.33 (6.01-6.65)
Transect	interior	5.6 (4.9-6.2)	11 (9.3-12)	8.3 (7.3-9.5)	0.66 (0.43-1.0)	4.7 (2.9-7.6)	**6.40 (6.20-6.60)**
	edge	5.4 (4.7-6.0)	10 (9.0-11)	7.9 (6.9-9.0)	0.69 (0.46-1.0)	5.7 (3.6-9.1)	**6.20 (6.01-6.40)**
Ploidy	2n	5.5 (4.5-6.4)	10 (8.9-12)	7.7 (6.5-9.2)	0.75 (0.40-1.4)	7.1 (3.3-15)	6.18 (5.85-6.50)
	4n	5.5 (4.6-6.4)	10 (8.8-12)	8.5 (7.3-9.9)	0.61 (0.34-1.1)	3.8 (1.8-7.7)	6.43 (6.13-6.72)
Field size		-0.24	**-0.088**	**-0.074**	**-0.21**	0.024	-0.032
Arable		-3.4	-0.37	**0.91**	-0.53	-1.3	0.31
Flowers		-0.0013	0.0065	**0.0098**	-0.015	-0.011	0.00069

Strip area	0.00035	0.000098	0.000090	0.000037	0.00039	-0.00025

Table S4. Densities (bees per 50 m transect and survey round) of all bumble bees and short tongued (<7 mm) and long tongued (>7 mm) bumble bees and honey bees during the time period when both the phacelia and clover flowered, in relation to year, region and ploidy (2n, diploid; 4n, tetraploid), field size, proportion of arable land in the 1 km circular landscape surrounding the clover field, flower density and, for fields with flower strip (n = 22), transect location (in the phacelia flower strip or in the adjacent edge or interior of the clover field) and, for all fields surveyed during the period (n = 44), transect location (edge or interior) and presence of flower strip. Numbers indicate least square mean values and the 95% confidence limits (cl) estimated from the statistical models, back-transformed using the ILINK option when needed, and the model-estimated slope for the continuous variables field size, arable land and flower density. Bold numbers are for significant effects where P <0.050 (see Table 2).

		Bumble bees<br><br>estimate (cl)	Short tongued<br>bumble bees<br>estimate (cl)	Long tongued<br>bumble bees<br>estimate (cl)	Honey bees<br><br>estimate (cl)
Fields with flower strip					
Year	2009	**5.7 (3.8-8.5)**	**4.7 (3.0-7.3)**	**0.31 (0.12-0.79)**	**2.2 (1.1-4.7)**
	2010	**1.9 (1.3-2.9)**	**1.6 (1.0-2.4)**	**0.082 (0.025-0.27)**	**0.72 (0.34-1.5)**
Region	Skåne	4.7 (3.0-7.2)	3.8 (2.3-6.1)	0.29 (0.11-0.78)	**2.9 (1.3-6.3)**
	Östergötland	2.3 (1.4-3.8)	2.0 (1.2-3.3)	0.087 (0.020-0.37)	**0.56 (0.22-1.4)**
Ploidy	2n	3.5 (2.2-5.7)	2.9 (1.8-5.0)	0.095 (0.021-0.43)	2.1 (0.87-4.9)
	4n	3.1 (2.0-4.8)	2.5 (1.6-4.0)	0.27 (0.10-0.71)	0.78 (0.33-1.8)
Field size		0.028	0.012	0.15	0.15
Arable		-0.032	0.10	1.9	**-2.7**
Flowers		**0.028**	**0.030**	0.020	**0.028**
Transect	interior	**1.8 (1.2-2.6)**	**1.4 (0.93-2.2)**	0.19 (0.068-0.54)	**0.11 (0.040-0.32)**
	edge	**1.5 (1.0-2.2)**	**1.2 (0.76-1.8)**	0.10 (0.031-0.32)	**0.89 (0.44-1.8)**
	phacelia	**14 (10-18)**	**12 (8.8-16)**	0.21 (0.076-0.60)	**20 (12-33)**
All fields					
Year	2009	**3.5 (2.6-4.6)**	**2.8 (2.1-3.9)**	**0.32 (0.19-0.56)**	**0.93 (0.50-1.7)**
	2010	**1.2 (0.81-1.7)**	**0.90 (0.60-1.4)**	**0.12 (0.050-0.28)**	**0.19 (0.078-0.48)**
Region	Skåne	**3.4 (2.6-4.6)**	**2.5 (1.8-3.5)**	**0.38 (0.22-0.64)**	0.76 (0.38-1.5)
	Östergötland	**1.2 (0.79-1.8)**	**1.0 (0.65-1.6)**	**0.10 (0.037-0.27)**	0.24 (0.092-0.61)
Ploidy	2n	2.3 (1.7-3.2)	1.8 (1.3-2.6)	0.25 (0.13-0.48)	0.47 (0.22-0.99)
	4n	1.7 (1.3-2.4)	1.4 (0.97-2.0)	0.15 (0.07-0.33)	0.38 (0.17-0.82)
Field size		-0.031	-0.069	0.051	0.085
Arable		-0.83	-0.68	0.19	0.31
Flowers		**0.015**	**0.018**	-0.0019	**0.027**
Transect	interior	2.2 (1.7-2.8)	1.8 (1.3-2.3)	0.19 (0.10-0.37)	**0.22 (0.11-0.43)**
	edge	1.9 (1.4-2.4)	1.4 (1.1-1.9)	0.20 (0.10-0.37)	**0.81 (0.46-1.4)**

Strip	no	2.4 (1.7-3.4)	1.9 (1.3-2.7)	0.16 (0.073-0.33)	0.47 (0.22-1.0)
	yes	1.7 (1.2-2.4)	1.4 (0.93-2.0)	0.24 (0.12-0.49)	0.38 (0.17-0.85)

Table S5. Density of *Protapion* spp. weevils (weevils per clover inflorescence), pest parasitism rate (proportion of weevil larvae attacked by a parasitoid) and the yield of clover seed (kg ha^{-1}) for the different years, regions, transects and ploidy (2n, diploid; 4n, tetraploid), in relation to the proportion of arable land in the 1 km circular landscape surrounding the clover field and presence of flower strip (including all 50 fields) or size of the flower strip (m^2) (including the 22 fields with flower strips). Numbers indicate least square mean values and the 95% confidence limits (cl) estimated from the statistical models, back-transformed using the ILINK option when needed, and the model-estimated slope for the continuous variables field size, arable land, flower density and flower strip area. Bold numbers are for significant effects where P <0.050 (see Table 3).

		Weevils estimate (cl)	Parasitism estimate (cl)	Seed yield estimate (cl)
All fields				
Year	2009	**1.1 (0.53-2.0)**	**0.045 (0.025-0.080)**	320 (240-400)
	2010	**0.16 (0.077-0.32)**	**0.20 (0.12-0.32)**	280 (210-360)
Region	Skåne	**0.81 (0.42-1.5)**	0.13 (0.082-0.20)	**410 (340-480)**
	Östergötland	**0.20 (0.090-0.46)**	0.075 (0.033-0.16)	**200 (110-280)**
Transect	interior	0.41 (0.25-0.69)	**0.077 (0.048-0.12)**	310 (260-370)
	edge	0.40 (0.24-0.66)	**0.13 (0.081-0.19)**	290 (240-350)
Ploidy	2n	**0.20 (0.093-0.41)**	**0.15 (0.088-0.25)**	**400 (330-480)**
	4n	**0.84 (0.40-1.8)**	**0.062 (0.031-0.12)**	**200 (120-280)**
Field size		-0.12	0.011	-0.12
Arable		-1.4	-1.9	-220
Flowers		-0.00016	0.014	1.8
Strip	no	0.38 (0.20-0.75)	0.13 (0.079-0.20)	310 (240-380)
	yes	0.42 (0.20-0.91)	0.074 (0.035-0.15)	290 (210-370)
Fields with flower strip				
Year	2009	**0.92 (0.39-2.2)**	**0.036 (0.015-0.082)**	330 (220-440)
	2010	**0.28 (0.13-0.63)**	**0.16 (0.076-0.30)**	260 (160-360)
Region	Skåne	**1.5 (0.61-3.5)**	0.10 (0.045-0.22)	**410 (290-530)**
	Östergötland	**0.18 (0.058-0.54)**	0.056 (0.015-0.19)	**180 (57-310)**
Transect	interior	0.51 (0.27-0.94)	**0.058 (0.030-0.11)**	310 (230-390)
	edge	0.51 (0.28-0.95)	**0.10 (0.055-0.18)**	280 (210-360)
Ploidy	2n	0.37 (0.13-1.0)	0.094 (0.033-0.24)	**400 (270-520)**
	4n	0.71 (0.27-1.9)	0.063 (0.020-0.18)	**200 (78-310)**
Field size		-0.14	0.14	-16
Arable		-1.3	-1.8	-310
Flowers		-0.013	**0.021**	**5.8**
Strip area		-0.0014	0.00068	**0.30**

References

Goulson, D., Hanley, M.E., Darvill, B., Ellis, J.S. & Knight, M.E. (2005) Causes of rarity in bumblebees. *Biological Conservation*, **122**, 1-8.

Kleijn, D., Winfree, R., Bartomeus, I., Carvalheiro, L.G., Henry, M., Isaacs, R., Klein, A.M., Kremen, C., M'Gonigle, L.K., Rader, R., Ricketts, T.H., Williams, N.M., Adamson, N.L., Ascher, J.S., Baldi, A., Batary, P., Benjamin, F., Biesmeijer, J.C., Blitzer, E.J., Bommarco, R., Brand, M.R., Bretagnolle, V., Button, L., Cariveau, D.P., Chifflet, R., Colville, J.F., Danforth, B.N., Elle, E., Garratt, M.P.D., Herzog, F., Holzschuh, A., Howlett, B.G., Jauker, F., Jha, S., Knop, E., Krewenka, K.M., Le Feon, V., Mandelik, Y., May, E.A., Park, M.G., Pisanty, G., Reemer, M., Riedinger, V., Rollin, O., Rundlof, M., Sardinas, H.S., Scheper, J., Sciligo, A.R., Smith, H.G., Steffan-Dewenter, I., Thorp, R., Tscharntke, T., Verhulst, J., Viana, B.F., Vaissiere, B.E., Veldtman, R., Westphal, C. & Potts, S.G. (2015) Delivery of crop pollination services is an insufficient argument for wild pollinator conservation. *Nature Communications*, **6**.

Murray, T.E., Fitzpatrick, U., Brown, M.J.F. & Paxton, R.J. (2008) Cryptic species diversity in a widespread bumble bee complex revealed using mitochondrial DNA RFLPs. *Conservation Genetics*, **9**, 653-666.

Persson, A.S., Rundlöf, M., Clough, Y. & Smith, H.G. (2015) Bumble bees show trait-dependent vulnerability to landscape simplification. *Biodiversity and Conservation*, **24**, 3469-3489.

Pywell, R.F., Warman, E.A., Carvell, C., Sparks, T.H., Dicks, L.V., Bennett, D., Wright, A., Critchley, C.N.R. & Sherwood, A. (2005) Providing foraging resources for bumblebees in intensively farmed landscapes. *Biological Conservation*, **121**, 479-494.
